# Gestational weight gain during the second and third trimesters and adverse pregnancy outcomes, results from a prospective pregnancy cohort in urban Tanzania

**DOI:** 10.1186/s12978-022-01441-7

**Published:** 2022-06-16

**Authors:** Jiaxi Yang, Molin Wang, Deirdre K. Tobias, Janet W. Rich-Edwards, Anne Marie Darling, Ajibola I. Abioye, Andrea B. Pembe, Isabel Madzorera, Wafaie W. Fawzi

**Affiliations:** 1 Department of Epidemiology, Harvard T. H. Chan School of Public Health, 677 Huntington Ave, Boston, MA 02115 USA; 2 Global Center for Asian Women’s Health, Yong Loo Lin School of Medicine, National University of Singapore, Singapore, Singapore; 3 Department of Obstetrics and Gynaecology, Yong Loo Lin School of Medicine, National University of Singapore, Singapore, Singapore; 4 Department of Biostatistics, Harvard T. H. Chan School of Public Health, 677 Huntington Ave, Boston, MA 02115 USA; 5 Department of Nutrition, Harvard T. H. Chan School of Public Health, 677 Huntington Ave, Boston, MA 02115 USA; 6 Division of Preventive Medicine, Department of Medicine, Brigham and Women’s Hospital, 900 Commonwealth Avenue, Boston, MA 02115 USA; 7 Channing Division of Network Medicine, Division of Women’s Health, Department of Medicine, Brigham and Women’s Hospital and Harvard Medical School, 900 Commonwealth Avenue, Boston, MA 02115 USA; 8 Department of Global Health and Population, Harvard T.H. Chan School of Public Health, 677 Huntington Ave, Boston, MA 02115 USA; 9 Department of Obstetrics and Gynecology, School of Medicine, Muhimbili University of Health and Allied Sciences, P. O. Box 65117, Dar es Salaam, Tanzania

**Keywords:** Gestational weight gain, Adverse birth outcomes, Tanzania, Institute of Medicine (U.S.)

## Abstract

**Background:**

Appropriate gestational weight gain (GWG) is important for optimal pregnancy outcomes. This study prospectively evaluated the associations between GWG during the second and third trimesters of pregnancy and adverse pregnancy outcomes in an urban Tanzanian pregnancy cohort.

**Methods:**

We used data from a randomized clinical trial conducted among pregnant women recruited by 27 weeks of gestation in Dar es Salaam, Tanzania (N = 1230). Women’s gestational weight was measured at baseline and at monthly antenatal visits. Weekly GWG rate during the second and third trimesters was calculated and characterized as inadequate, adequate, or excessive, in conjunction with measured or imputed early-pregnancy BMI status according to the 2009 Institute of Medicine (IOM) GWG guidelines. We used multivariable Poisson regression with a sandwich variance estimator to calculate risk ratios (RR) for associations of GWG with low birth weight, preterm birth, small for gestational age (SGA), and large for gestational age (LGA). Degree of appropriate GWG defined using additional metrics (i.e., percentage of adequacy, z-score) and potential effect modification by maternal BMI were additionally evaluated.

**Results:**

According to the IOM guidelines, 517 (42.0%), 270 (22.0%), and 443 (36.0%) women were characterized as having inadequate, adequate, and excessive GWG, respectively. Overall, compared to women with adequate GWG, women with inadequate GWG had a lower risk of LGA births (RR = 0.54, 95% CI: 0.36–0.80) and a higher risk of SGA births (RR = 1.32, 95% CI: 0.95–1.81). Women with inadequate GWG as defined by percentage of GWG adequacy had a higher risk of LBW (OR = 1.93, 95% CI: 1.03–3.63). In stratified analyses by early-pregnancy BMI, excessive GWG among women with normal BMI was associated with a higher risk of preterm birth (RR = 1.59, 95% CI: 1.03–2.44).

**Conclusions:**

A comparatively high percentage of excessive GWG was observed among healthy pregnant women in Tanzania. Both inadequate and excessive GWGs were associated with elevated risks of poor pregnancy outcomes. Future studies among diverse SSA populations are warranted to confirm our findings, and clinical recommendations on optimal GWG should be developed to promote healthy GWG in SSA settings.

*Trial registration:* This trial was registered as “Prenatal Iron Supplements: Safety and Efficacy in Tanzania” (NCT01119612; http://clinicaltrials.gov/show/NCT01119612).

**Supplementary Information:**

The online version contains supplementary material available at 10.1186/s12978-022-01441-7.

## Plain language summary

Pregnancy is a critical lifetime event for both mother and the offspring, with implications in short-term and long-term health consequences. Gestational weight gain (GWG) is an important modifiable factor for pregnancy outcomes related to infant body size and weight and prematurity. Countries in sub-Saharan Africa (SSA) have long had poor rates of insufficient GWG and pregnancy complications associated with insufficient GWG. Nevertheless, some SSA countries are experiencing economic transitions accompanied with changes in lifestyle and nutrition, which might impact pregnancy experiences, including GWG and pregnancy outcomes. This study aimed to characterize recent GWG patterns and the associations of both inadequate and excessive GWG with adverse pregnancy outcomes, using an urban pregnancy cohort in Tanzania. This study found that 42.0%. 22.0%, and 36.0% of women had insufficient, adequate, and excessive GWG, respectively. Insufficient GWG was associated with higher risks of small infant size and low infant body weight, and excessive GWG was associated with higher risk of preterm birth, particularly among women with body mass index 18.5–25.0 kg/m^2^. Results from the present study highlight that both insufficient and excessive GWG are of potential public health concerns in urban centers of SSA, concerning upward trends in obesity and possibly obesity-related pregnancy consequences. Local public health practitioners should continue to advocate longitudinal GWG monitoring and care among African pregnant women, and optimal GWG with feasible and effective clinical guidelines should be developed to prevent both over- and under-gaining of maternal weight during pregnancy.

**Supplementary Information:**

The online version contains supplementary material available at 10.1186/s12978-022-01441-7.

## Background

Pregnancy is a key health-related event for both the mother and the offspring. Women who developed pregnancy complications have higher risk of future pregnancy complications [1] and poor long-term health outcomes, including obesity [2], and metabolic [3] and cardiovascular diseases [4, 5]. Addressing poor birth outcomes is a critical step in improving child survival at a global scale [6]. Adverse birth outcomes, including prematurity and inappropriate intrauterine growth, have been associated with neonatal complications, higher neonatal and infant mortality [7], as well as life-long health and developmental problems in the offspring [8]. Therefore, identifying and intervening on factors associated with adverse pregnancy outcomes remains a critical means of addressing and preventing pregnancy-related complications, particularly for low- and middle-income countries where the rates of pregnancy complications remain high [9, 10].

Gestational weight gain (GWG) is one of the key modifiable factors associated with birth outcomes [11, 12]. Components of GWG include body composition of the mother, weight of the fetus, placenta, and amniotic fluid [13]. GWG reflects the health status of the mother and the growth of the fetus [13]. Most of the maternal weight gain takes place after the first trimester of a pregnancy [14, 15], with GWG in the second and third trimesters contributing to about 80–90% of the total GWG [13]. Prospective studies conducted in Caucasian or Asian populations have supported the associations between GWG in the second and the third trimesters and pregnancy outcomes related to infant body weight and size and prematurity [13, 15, 16].

Distinct GWG patterns have been observed across world regions, with overall higher proportions of suboptimal GWG especially in populations from low-income countries such as in sub-Saharan Africa (SSA), a region that has long had high rates of poor birth outcomes [7, 17–19]. Nonetheless, patterns of GWG across different African contexts are changing and may have become more heterogenous than they were in the past. Some SSA countries, such as South Africa, Kenya, and Tanzania, have recently undergone transition from low-income to middle-income status, accompanied with changes in lifestyles, including better food security, dietary transitions, and reduced physical activity [20, 21]. These changes may have led to changes in maternal diets prior to and during pregnancy, affecting GWG patterns and the overall pregnancy experience for women in these regions [22]. For example, a recent study conducted in South Africa reported that the percentage of excessive GWG was as high as 55%, which was even higher than the rates in some developed countries [23].

While the associations between GWG and pregnancy outcomes have been well examined in other countries, evidence from SSA remains inadequate. Several studies in SSA examined these associations, but they were largely limited by retrospective or cross-sectional designs, insufficient measures of pregnancy weight, and inadequate adjustment for key confounders, including pre-pregnancy BMI [17]. Of particular importance, the noted nutrition transition underway in urban centers of SSA with rising rates of overweight and obesity is likely not reflected in earlier studies. Therefore, we sought to prospectively examine the associations between GWG in the second and third trimesters and adverse birth outcomes in a healthy pregnancy cohort in Tanzania.

## Methods

### Study population

We used data from a randomized clinical trial conducted in urban Tanzania. Details of this study have been described elsewhere [24, 25]. Briefly, from September 2010 to October 2012, a randomized trial on iron supplements was conducted in Dar es Salaam, Tanzania. Participants were screened and enrolled at antenatal care clinics. Women were eligible if they were iron-replete, non-anemic, HIV-uninfected, primigravidae or secundigravidae, and were recruited at or before 27 weeks of gestation. Baseline gestational age (weeks) was estimated based on the reported timing of the last menstrual period (LMP). The study enrolled 1500 pregnant women who were subsequently randomized to receive a daily oral dose of either 60 mg of iron or placebo from the time of enrollment until delivery.

At baseline, women completed a sociodemographic and reproductive health questionnaire, as well as a full clinical examination. They were subsequently followed at monthly antenatal visits and at time of delivery. For our study, we excluded participants with unknown gestational age at delivery (n = 22), unknown delivery outcomes (n = 15), or twin babies (n = 27). Since GWG in the second and third trimesters was the main exposure of interest, we further excluded women with only one weight measure during that time window (n = 206), leaving us with a final study sample of 1230 participants.

### Assessment and characterization of GWG

Study participants’ weight at baseline and at monthly follow-up visits was measured by trained study nurses using a calibrated weight scale. Pre-pregnancy BMI has been suggested as an important covariate for the association between GWG and pregnancy outcomes [13]. However, information on pre-pregnancy weight was not collected in the original trial study. Further, given the distribution of the baseline gestational age at enrollment, only 196 out of 1230 participants were enrolled during the first trimester, of which the majority were enrolled in the late first trimester (interquartile range of gestational age at enrollment among participants enrolled in the first trimester: 10–13 weeks). We therefore imputed BMI at the end of the first trimester (14 weeks of gestation) for covariate adjustment and stratification. Based on the repeated weight measurements during pregnancy, we fit mixed-effects models with polynomial terms of gestational age (weeks) and imputed individual-specific weight at 14 weeks of gestation; statistical results suggested good imputation performance (mean absolute error: 1.95 kg, concordance rate of categorical BMI among women with available first-trimester weight: 89.0%). Details on the statistical methods and the imputation results can be found elsewhere [25]. Based on the imputed weight at the end of the first trimester and the height measured at baseline, the corresponding BMI status at the end of the first trimester was derived (underweight if BMI < 18.5 kg/m^2^, normal if 18.5 kg/m^2^ ≤ BMI < 25 kg/m^2^, overweight if 25 kg/m^2^ ≤ BMI < 30 kg/m^2^, and obese if BMI ≥ 30 kg/m^2^).

The main exposure of interest was GWG during the second and third trimesters. We defined degree of appropriate GWG based on the 2009 Institute of Medicine (IOM) guidelines [13]. The IOM guidelines provided recommended ranges for total weight gain and the rate of weight gain during the second and third trimesters, based on pre-pregnancy BMI status. Weekly rate of GWG during the second and third trimesters (kg/week) was derived by calculating the difference between the first measured weight in the second trimester and the last measured weight before delivery and dividing that by the number of weeks between the two measures. For each given participant, based on the calculated weekly rate of GWG, the BMI status at the end of the first trimester, and the IOM recommended GWG range (0.44–0.58 kg/week for underweight, 0.35–0.50 kg/week for normal weight, 0.23–0.33 kg/week for overweight, and 0.17–0.27 kg/week for obese), GWG was characterized as inadequate (weekly rate of GWG below the recommended range), adequate (weekly rate of GWG within the recommended range), or excessive (weekly rate of GWG above the recommended range) [13]. We made assumptions that weight gain during the first trimester was minimal and that women stayed in the same BMI category from the start of the pregnancy until the end of the first trimester [13].

We additionally characterized GWG using other metrics, including percentage of GWG adequacy (i.e. percentage method) [26] and GWG z-score (i.e., z-score method) based on the INTERGROWTH-21st standard [27]. Building upon the IOM guidelines which grouped the extent of GWG into three categories (i.e., inadequate, adequate, and excessive GWG), the percentage method provided a percentage value to further quantify the amount of GWG relative to the guidelines with accounting for the pregnancy duration. Details on this method has been described elsewhere [26]. Briefly, percentage adequacy of GWG was calculated as the ratio of observed weight gain (kg) and expected weight gain (kg) during pregnancy. The original formula was given as follows: percent adequacy = observed weight gain during pregnancy / [expected first trimester weight gain + ((week at the last weight measure – 13)*expected weekly GWG rate in the second and third trimesters)]. Given the research question of our study, we modified the formula by restricting the time period to the second and third trimesters instead of the entire pregnancy. Consistent with Adu-Afarwuah et al., we derived BMI-specific percentage cutoffs based on the IOM cutoffs and classified the GWG into three groups based on the calculated percent adequacy: inadequate, adequate, and excessive, respectively [26].

We further constructed a GWG z-score (in unit of standard deviation) for participants with a normal BMI (18.5 kg/m^2^ ≤ BMI < 25 kg/m^2^) at the end of the first trimester, using a standard reference chart developed by the INTERGROWTH-21st consortium [27]. Briefly, by applying the reference chart, a gestational age-specific GWG z-score can be derived based on the total weight (kg) gained up to a given gestational age. Since GWG was likely to follow a non-linear trajectory over the course of pregnancy, a gestational age-specific z-score could account for the natural correlation between a longer pregnancy duration and a higher rate of GWG [28], which, if unaddressed, could bias the association between GWG and gestational age-related outcome (e.g., prematurity). For our analysis, total weight gain in the second and third trimesters and gestational age at the last weight measure were used to derive the z-score. Given the potential non-linearity of the z-score with respect to risks of pregnancy outcomes and the distribution of the z-scores in our sample (only one participant had z-score > 2 units), we classified participants into one of the two following groups: inadequate GWG if z-score < -2 units (2.3th percentile), adequate GWG if z-score within ± 2 units (between 2.3th and 97.7th percentile).

### Outcome assessment

At the time of delivery, on-site midwives recorded participants’ pregnancy outcomes. Data on gestational age at delivery (weeks), delivery outcome if known (miscarriage [n = 1], stillbirth [n = 47], and live birth [n = 1182]), infant sex, and infant birth weight (kg) were determined. We derived the following outcome variables for pregnancies resulting in live births: low birth weight (LBW, birthweight < 2.5 kg), preterm birth (gestational age at delivery < 37 weeks), small for gestational age and large for gestational age (SGA and LGA, gender-specific birth weight below the 10^th^ percentile and above the 90^th^ percentile respectively for babies of the same gestational age according to the INTERGROWTH-21st reference) [29]. Although we did not have information on the type of preterm birth (i.e., spontaneous, medically induced), we considered most of the preterm cases as spontaneous, based on conversations with on-site research staff and medically induced preterm birth being relatively uncommon in Tanzania.

### Statistical analysis

In the main analyses, GWG during the second and third trimesters according to the IOM recommendations was evaluated with respect to adverse birth outcomes. GWG with three levels defined by the IOM guidelines (i.e., inadequate, adequate, and excessive GWG) was modeled as a categorical variable, and the group with adequate GWG was set as the reference group. The following dichotomous outcomes were examined: LBW, preterm birth, SGA, and LGA. We used multivariable Poisson regression with a sandwich variance estimator to estimate risk ratio (RR) and 95% confidence interval (CI) [30]. We adjusted for covariates hypothesized a priori as potential confounders in the analyses, including age, baseline gestational age, gestational age at delivery, measured or imputed BMI at 14 weeks of gestation, primigravida status, treatment status, marital status, education, occupation, and history of prior complications (history of cardiovascular disease, high blood pressure, diabetes, or weight loss in previous year, or ever had a LBW baby or non-live birth among non-primigravida).

Given the evidence of the heterogeneity by pre-pregnancy BMI status for the associations of interest [13], we further stratified the analyses by BMI status at the end of the first trimester. Due to the limited sample size, we did not examine these associations among underweight women (n = 72) and examined the questions among women with normal BMI (18.5 kg/m^2^ ≤ BMI < 25 kg/m^2^; n = 756) and overweight or obese women (BMI ≥ 25 kg/m^2^; n = 402 [295 and 107 for overweight and obese, respectively]), separately. Heterogeneity was evaluated by the statistical significance of the cross-product term between categorical GWG and BMI status in the analysis sample excluding underweight women.

In the sensitivity analyses, we additionally examined appropriate GWG and birth outcomes, using the percentage and the z-score methods. For the z-score method, since the INTERGROWTH-21^st^ GWG reference chart is currently available only for women with normal pre-pregnancy BMI, we restricted the analyses to participants with normal BMI at the end of the first trimester (n = 755). All analyses were conducted using SAS statistical software (version 9.4; SAS Institute Inc, Cary, NC, USA). All statistical tests were 2-sided, with *p*-value less than 0.05 considered as statistically significant.

## Results

Our analysis included 1,230 women, with a mean baseline gestational age of 17.9 weeks (Table 1). According to the IOM recommendations, 517 (42.0%), 270 (22.0%), and 443 (36.0%) women had inadequate, adequate, and excessive GWG, respectively. Compared to women with adequate GWG, women with inadequate GWG were more likely to be unemployed and report a prior history of complications. Women with excessive GWG were more likely to be primigravida and have a higher early-pregnancy BMI, better educational status, and skilled occupation (Table 1).

**Table 1:** Study population baseline characteristics overall and by status of GWG according to the 2009 IOM guidelines

Baseline characteristics	Entire dataset (n = 1230)	2009 IOM GWG guidelines^a^		
		Inadequate GWG (n = 517, 42.0%)	Adequate GWG (n = 270, 22%)	Excessive GWG (n = 443, 36%)
*Mean (SD)*				
Age at baseline (years)	24.1 (4.2)	23.9 (4.4)	24.1 (3.8)	24.2 (4.2)
Weight at baseline	59.9 (11.7)	59.1 (12.0)	58.3 (10.5)	61.8 (11.8)
Height at baseline (cm)	156.2 (6.1)	156.0 (6.3)	155.8 (5.8)	156.7 (5.9)
Gestational age at baseline (weeks)	17.9 (4.3)	18.1 (4.5)	17.5 (4.2)	18.0 (4.1)
Total number of antenatal visits^b^	5 (2–9)	5 (2–8)	5 (2–8)	5 (2–9)
*n (%)*				
Treatment (iron supplement)	601 (48.9)	247 (47.8)	137 (50.7)	221 (49.9)
Primigravida	706 (57.4)	285 (55.1)	150 (55.6)	271 (61.2)
Marital status (married)	979 (79.6)	420 (81.2)	219 (81.1)	345 (77.9)
BMI at baseline (kg/m^2^)	24.5 (4.6)	24.3 (4.7)	24.1 (4.2)	25.1 (4.5)
BMI at 14 weeks of gestation (kg/m^2^)	24.0 (4.3)^d^	23.5 (4.4)	23.5 (4.0)	24.9 (4.4)
Education status				
0–4 years	61 (5.0)	25 (4.8)	14 (5.2)	22 (5.0)
5–7 years	645 (52.4)	286 (55.3)	147 (54.4)	212 (47.9)
8–11 years	343 (27.9)	136 (26.3)	75 (27.8)	132 (29.8)
≥ 12 years	181 (14.7)	70 (13.5)	34 (12.6)	77 (17.4)
Occupation status				
Unemployed	619 (50.3)	274 (53.0)	134 (49.6)	211 (47.6)
Unskilled or informal	381 (31.0)	154 (29.8)	89 (33.0)	138 (31.2)
Skilled	230 (18.7)	89 (17.2)	47 (17.4)	94 (21.2)
History of prior complications^c^	1–91 (15.5)	49 (18.2)	78 (15.1)	64 (14.5)

Institute of Medicine (IOM), gestational weight gain (GWG), standard deviation (SD), body mass index (BMI)

^a^The IOM provided recommended ranges of weekly GWG rate during the 2nd and 3rd trimesters (kg/week) by pre-pregnancy BMI status: 0.44––0.58 kg/week for underweight, 0.35–0.50 kg/week for normal weight, 0.23–0.33 kg/week for overweight, and 0.17–0.27 kg/week for obese. BMI categories were defined according to the WHO standard BMI guidelines

^b^Median (interquartile range) were presented

^c^History of prior complications was defined as reporting any of the following: cardiovascular disease, high blood pressure, diabetes, weight loss in previous year, ever having a low-birth-weight baby or non-live birth (fetal death, abortion, miscarriage, ectopic pregnancy) among non-primigravida

^d^Imputed gestational weight at 14 weeks of gestation

GWG-related characteristics and pregnancy outcomes for the entire sample and by the IOM-defined GWGs were summarized in Table 2. Classifications of appropriate GWG by the IOM guidelines and the percentage method were overall consistent: 22.0% were classified as adequate GWG using the IOM method, compared with 30.6% using the percentage method. Yet, compared to the GWG defined by the IOM recommendations, the percentage method was slightly more conservative, as it classified more women having inadequate or adequate GWG and fewer women having excessive GWG. Among women with normal BMI at the end of the first trimester (n = 756), mean GWG z-score was -1.9 (SD = 1.5); 428 (56.7%) and 327 (43.3%) had z-score within ± 2 and below -2 units of the z-score, respectively, with only one subject having a z-score above 2 units. With respect to the pregnancy outcomes, a total of 92 cases of LBW (7.5%), 195 cases of preterm birth (15.9%), 199 cases of SGA (16.2%), 134 cases of LGA (10.9%), and 47 cases of stillbirth (3.8%), were observed (Table 2).

**Table 2:** Summary characteristics of GWG and pregnancy outcomes in the study population overall and by GWG status

Outcomes	Entire dataset (n = 1230)	2009 IOM GWG guidelines^a^		
		Inadequate GWG (n = 517, 42.0%)	Adequate GWG (n = 270, 22.0%)	Excessive GWG (n = 443, 36.0%)
GWG-related outcomes				
Weight gain (kg), mean (SD)	6.3 (4.9)	2.8 (3.4)	6.9 (2.8)	10.1 (4.4)
Rate of weight gain in 2nd–3rd trimester (kg/week), mean (SD)	0.38 (0.32)	0.14 (0.21)	0.39 (0.09)	0.65 (0.28)
GWG adequacy, n (%)^b^				
Inadequate GWG	553 (45.0)	456 (88.2)	82 (30.4)	15 (3.4)
Adequate GWG	377 (30.6)	48 (9.3)	174 (64.4)	155 (35.0)
Excessive GWG	300 (24.4)	13 (2.5)	14 (5.2)	273 (61.6)
Adverse pregnancy outcomes				
Gestational age at delivery (weeks), mean (SD)	39.5 (3.3)	39.7 (3.6)	39.6 (2.7)	39.0 (3.3)
Infant birth weight (kg), mean (SD)	3.1 (0.5)	3.1 (0.5)	3.2 (0.5)	3.2 (0.6)
Low birth weight (< 2.5 kg), n (%)	92 (7.5)	40 (7.7)	15 (5.6)	37 (8.4)
Preterm birth (< 37 weeks), n (%)	195 (15.9)	76 (14.7)	41 (15.2)	78 (17.6)
SGA, n (%)^c^	199 (16.2)	101 (19.5)	40 (14.8)	58 (13.1)
LGA, n (%)^c^	134 (10.9)	36 (7.0)	38 (14.0)	60 (13.5)
Stillbirth, n (%)^d^	47 (3.8)	12 (2.3)	9 (3.3)	26 (5.9)

Institute of Medicine (IOM), gestational weight gain (GWG), standard deviation (SD), small for gestational age (SGA), large for gestational age (LGA)

^a^The IOM provided recommended ranges of weekly GWG rate during the 2nd and 3rd trimesters (kg/week) by pre-pregnancy BMI status: 0.44–0.58 kg/week for underweight, 0.35–0.50 kg/week for normal weight, 0.23–0.33 kg/week for overweight, and 0.17–0.27 kg/week for obese. BMI categories were defined according to the WHO standard BMI guidelines

^b^GWG adequacy was calculated based on the method described in Adu-Afarwuah, Seth, et al. "Maternal supplementation with small-quantity lipid-based nutrient supplements compared with multiple micronutrients, but not with iron and folic acid, reduces the prevalence of low gestational weight gain in semi-urban Ghana: a randomized controlled trial." The Journal of nutrition 147.4 (2017): 697–705

^c^For babies of the same gestational age (gender-specific), birthweight below the 10th percentile and above the 90th percentile was defined as SGA and LGA, respectively, based on the INTERGROWTH-21st reference chart

^d^Stillbirth was defined as fetal death at or after 20 weeks of gestation

In the main analyses, compared to the reference group with adequate GWG, women who had inadequate GWG experienced a lower risk of LGA (RR = 0.54, 95% CI: 0.36–0.80) and a higher risk of SGA (RR = 1.32, 95% CI: 0.95–1.81). For the group of excessive GWG, compared to the reference group, no significant difference in risks was observed across the outcomes that we examined, including LBW, preterm birth, SGA, or LGA (Table 3).

**Table 3:** Associations between GWG by the IOM and adverse pregnancy outcomes overall and stratified by BMI status

End of 1st trimester BMI	2009 IOM guidelines^b^	Pregnancy outcomes, risk ratio (95% CI)^a^			
		LBW^c^	Preterm birth^d^	SGA	LGA
	Cases (n, percent)^e^	92, 7.5%	195, 15.9%	199, 16.2%	134, 10.9%
Total (N = 1230)	Inadequate GWG (n = 517, 42.0%)	1.30 (0.67, 2.54)	0.99 (0.70, 1.40)	1.32 (0.95, 1.81)	0.54 (0.36, 0.80)
	Adequate GWG (n = 270, 22.0%)	Ref (OR = 1.00)	Ref (RR = 1.00)	Ref (RR = 1.00)	Ref (RR = 1.00)
	Excessive GWG (n = 443, 36.0%)	1.27 (0.63, 2.53)	1.22 (0.86, 1.73)	0.96 (0.67, 1.38)	0.89 (0.62, 1.29)
	Cases (n, percent)	66, 8.7%	124, 16.4%	130, 17.2%	82, 10.8%
Normal 18.5 ≤ BMI < 25 (N = 756)	Inadequate GWG (n = 343, 45.4%)	1.38 (0.63, 3.06)	1.20 (0.79, 1.84)	1.29 (0.89, 1.89)	0.65 (0.39, 1.07)
	Adequate GWG (n = 189, 25.0%)	Ref (OR = 1.00)	Ref (RR = 1.00)	Ref (RR = 1.00)	Ref (RR = 1.00)
	Excessive GWG (n = 224, 29.6%)	1.17 (0.50, 2.72)	1.59 (1.03, 2.44)	0.96 (0.61, 1.50)	1.31 (0.83, 2.06)
	Cases (n, percent)	16, 4.0%	59, 14.7%	53, 13.2%	50, 12.4%
Overweight or obese BMI ≥ 25 (N = 402)	Inadequate GWG (n = 132, 32.8%)	0.54 (0.10, 2.85)	0.79 (0.40, 1.58)	1.10 (0.55, 2.18)	0.34 (0.17, 0.70)
	Adequate GWG (n = 65, 16.2%)	Ref (OR = 1.00)	Ref (RR = 1.00)	Ref (RR = 1.00)	Ref (RR = 1.00)
	Excessive GWG (n = 205, 51.0%)	0.87 (0.21, 3.69)	0.85 (0.46, 1.57)	0.87 (0.44, 1.72)	0.45 (0.25, 0.80)
	*P*-heterogeneity^f^	0.97	0.64	0.97	0.01

Institute of Medicine (IOM), gestational weight gain (GWG), body mass index (BMI), low birth weight (LBW), small for gestational age (SGA), large for gestational age (LGA), odds ratio (OR), risk ratio (RR), confidence interval (CI)

^a^Multivariable model was adjusted for age (years), baseline gestational age (weeks), gestational age at delivery (weeks), BMI at 14 weeks of gestation (underweight, normal, overweight, obese), primigravida status (yes, no), treatment status (iron, placebo), marital status (married, other than married), education (0–4 years, 5–7 years, 8–11 years, ≥ 12 years), occupation (unemployed, unskilled or informal, skilled), and history of prior complications (yes, no)

^b^The IOM provided recommended ranges of weekly GWG rate during the 2nd and 3rd trimesters (kg/week) by pre-pregnancy BMI status: 0.44–0.58 kg/week for underweight, 0.35–0.50 kg/week for normal weight, 0.23—0.33 kg/week for overweight, and 0.17—0.27 kg/week for obese

^c^Model for estimating RR did not converge due to small number of LBW events; OR from multivariable logistic regression was reported to approximate RR instead

^d^Gestational age at delivery was not adjusted in the model for preterm birth

^e^Total number and percent of cases in overall and in groups of normal BMI and overweight/obesity were presented

^f^*P*-value for heterogeneity was computed for the interaction term between GWG and BMI status at the end of 1st trimester excluding underweight women

In the stratified analyses by BMI at the end of the first trimester, excessive GWG was associated with a higher risk of preterm birth (RR = 1.59, 95% CI: 1.03–2.44) among women with normal BMI. Among women who were overweight or obese, no elevated risk was observed with either inadequate GWG or excessive GWG compared with the reference group. In fact, a lower risk of LGA was observed in both groups of inadequate GWG (RR = 0.34, 95% CI: 0.17–0.70) and excessive GWG (RR = 0.45, 95% CI: 0.25–0.80). Significant statistical differences were observed between the groups with normal BMI and overweight or obesity for LGA (*p*-heterogeneity = 0.01) (Table 3).

Additional analyses using the two other GWG metrics were largely consistent with the main findings. Results from the percentage GWG method suggested that, inadequate GWG was associated with higher risks of LBW (OR = 1.93, 95% CI: 1.03–3.63) and SGA (RR = 1.53, 95% CI: 1.14–2.07) and a lower risk of LGA (RR = 0.53, 95% CI: 0.38–0.77). Similar to the main analyses, overall, no significant associations were observed between excessive GWG and the outcomes examined (Additional file 1: Table S1). Among women with normal BMI at the end of the first trimester, compared to the results using the IOM classifications, results using the z-score method showed similar directions of the associations, overall (Additional file 1: Table S2).

## Discussion

This study prospectively examined GWG during the second and third trimesters and adverse pregnancy outcomes in an urban pregnancy cohort with singleton births in Tanzania. We modeled GWG during the second and third trimesters of pregnancy using multiple metrics in conjunction with birth outcomes. We found that inadequate GWG was associated with a lower risk of LGA and higher risks of SGA and LBW, and excessive GWG was associated with higher risk of preterm birth, particularly among women with normal BMI.

Overall, studies have suggested heterogeneity in GWG across different SSA countries, with rising rates of excessive GWG reported in the countries of higher economic status, compared with other countries in the region [17]. In this study including healthy women in urban Tanzania, more than a third of women were found to have inadequate GWG, and a similar proportion had excessive GWG during the second and third trimesters of pregnancy. These GWG characteristics reported are in slight contrast to those reported from studies in SSA countries. A meta-analysis by Asefa et al. recently reviewed GWG in SSA according to the IOM guidelines and found that out of the sixteen SSA studies that they examined, nine studies had more than half of pregnant women with inadequate GWG. However, four studies from lower-middle-income or middle-income countries included in the review reported higher prevalence of excessive GWG (30.6% and 32.0% from two urban studies in Cameroon, 55.5% from an urban study in South Africa, and 29.6% from a clinic-based study in South Africa) [17]. Our findings are thereby consistent with reported rising tide of excessive GWG in SSA populations with lower-middle or middle-income status.

Prior studies in SSA have mainly focused on examining inadequate GWG given prevailing concerns for undernutrition in many countries in Africa [31]. In line with the overall literature evidence [17, 32], we reported that inadequate GWG was associated with higher risks of SGA and LBW and lower risk of LGA among pregnant women in urban Tanzania. The meta-analysis on GWG and pregnancy outcomes in SSA noted earlier reported overall significant associations between lower GWG and higher risk of LBW [17]. An association between inadequate GWG and lower risk of LGA observed in our study has also been supported in prospective studies of SSA [32] or other middle-income countries [33]. Finally, Johnson et al. prospectively examined GWG as defined by the INTERGROWTH-21st standards, and they reported that greater GWG was associated with lower risk of SGA (RR = 0.58, 95% CI: 0.46–0.72), consistent with the association found in our study [34].

While excessive GWG has been linked with higher risk of LGA and lower risks of LBW and SGA in other populations [33, 35–37], the relationships between excessive GWG and these outcomes in SSA have not been adequately examined. Limited evidence from retrospective or small-scale studies have suggested lower rate of LBW among African women with greater GWG [38–40]. A recent observational study including 170,428 pregnancies from Lebanon retrospectively examined the association between GWG and risks of SGA and LGA (percentage of SGA and LGA: 8.5 and 9.6%, respectively); the authors reported that excessive GWG was related to lower risk of SGA and higher risk of LGA across BMI categories [32]. In this study, we did not observe an association between excessive GWG and elevated risk of LGA or reduced risk of SGA or LBW among women in Tanzania. It was possible that the amount of GWG difference between excessive vs. adequate GWG groups in our study may not be sufficient enough to detect a significant difference in outcome risks. Further, with a smaller sample size compared to the Lebanon study, our study was likely underpowered. Future studies with a greater range of GWG are needed to further evaluate excessive GWG with pregnancy outcomes among African women and confirm these findings.

For the outcome of preterm birth, while no difference in the risks was observed comparing inadequate and adequate GWG groups, a higher risk was seen in the group with excessive GWG, particularly among women with normal BMI in early pregnancy. So far, literature has presented evidence on both insufficient and excessive GWGs on higher risk of preterm birth (i.e., a U-shaped relationship) [41–44]. There were a few prospective or large-scale studies that examined GWG and preterm birth among African women. One study from Malawi (n = 1287) did not find significant difference in risks of preterm birth across the three GWG groups [45]. Another study among HIV-infected women in South Africa (n = 471) reported higher GWG and increased risk of spontaneous preterm birth (OR = 4.35, 95% CI: 1.55–12.21 for 1 kg/week increase of GWG) [46]. The earlier reported study in Lebanon also reported results supporting the U-shaped relationship between GWG and risk of preterm birth [32]. Furthermore, studies have demonstrated the association varied by pre-pregnancy BMI, with excessive GWG associated with a higher risk of preterm birth for women with greater BMI [42, 44]. Therefore, different findings across different studies may likely be due to differences in population characteristics, particularly pre-pregnancy BMI [47], different types of preterm births being examined (i.e., spontaneous vs. medically induced) [42], and failure to fully account for the correlation between GWG and gestational age [28].

GWG has long been considered as a critical marker for various in-pregnancy nutritional and physiological conditions [37, 48]. For the mechanisms of GWG and outcomes related to infant weight or size (i.e., LBW, SGA, and LGA) and prematurity, maternal weight gain reflects the health status of the mother and the growth of the fetus [13, 49]. Poor nutritional status, including macronutrient and micronutrient deficiencies, reduced immune function, and underlying maternal infection, might lead to inadequate GWG and smaller fetus growth, thus increasing the risks of LBW [50] and SGA [51]. Above factors and poor plasma volume expansion are also underlying causes for prematurity [52]. On the other hand, overnutrition [53] or impaired glucose function of the mother [54] can lead to excessive GWG, thus resulting in greater fetal growth and consequently higher risk of LGA. For the outcome of prematurity, excessive GWG may reflect underlying infection leading to increased nutrient requirement, which, if unmet, could result in preterm delivery [48]. Furthermore, studies have proposed a link between excessive GWG and higher risk of preterm birth through mechanisms related to pro-inflammatory response [41].

Compared to other countries, countries in SSA have long had poor rates of SGA, LBW, and prematurity, all of which have serious long-term health consequences [7, 55]. Affected newborns face neonatal and future complications, including cognitive impairment, poor growth and stunting, and noncommunicable diseases [31, 56]. On the other hand, given the rising trends in overweight and obesity, particularly among women in SSA countries experiencing transitions in economic status and dietary and nutrition transitions [20, 21], rates of excessive GWG and obesity-related pregnancy outcomes are also expected to rise. Such pregnancy complications have consequences in future obesity-related and metabolic complications for both mother and the offspring [4]. Therefore, clinical guidelines on maternal care should now evolve to start to monitor and emphasize both inadequate and excessive GWGs in SSA countries experiencing these transitions, in effort to prevent short-term and long-term pregnancy complications. Additionally, tailoring nutrition guidelines for women based on their pregnancy BMI and focusing on both underweight and overweight in nutrition counselling for women may be beneficial. We also observed that women who had excessive GWG were more likely to be primigravida. Given the observed patterns of GWG differed by parity status (i.e., primigravida, secundigravidae), future well-powered studies are needed to examine the associations of GWG and adverse pregnancy outcomes by parity status and other maternal baseline characteristics; and to evaluate whether GWG recommendations may be adjusted according to key maternal characteristics. Lastly, given the important role of maternal diets on GWG and pregnancy outcomes and that there is likely heterogeneity in dietary patterns by region, future studies should characterize regional dietary patterns and examine GWG and pregnancy outcomes in conjunction with maternal dietary data.

Monitoring GWG is an important step for addressing inappropriate GWG and preventing its negative consequences [12, 57]. However, current evidence suggests a general lack of longitudinal monitoring system of GWG in countries of SSA [18]. Therefore, local public health practitioners should identify effective and feasible strategies integrating GWG monitoring into routine antenatal care. Further, intervention trials are needed to evaluate factors associated with optimal GWG, such as diet and physical activity [58], and aim to develop feasible programs and provide clinically useful guidelines on GWG management for pregnant women in SSA.

This present study has several strengths, including using repeated measures on pregnancy weights, characterizing GWG by multiple metrics, prospectively examining the associations between GWG and pregnancy-related outcomes with detailed covariate adjustment. Of particular importance, while past observational studies often used baseline BMI varying across study subjects when examining the associations, we adjusted and stratified the analyses by early-pregnancy BMI that was anchored at the same gestational age with the use of statistical models, leading to higher efficiency in covariate adjustment and better accuracy in stratification, compared to earlier SSA studies.

This study has some limitations. First, classification of appropriate GWG based on the IOM guidelines required the knowledge of pre-pregnancy BMI, which was not available in the study. Instead, we used BMI at the end of the first trimester to characterize GWG, which may lead to exposure misclassification. However, because BMI at the end of the first trimester was constructed as a categorical variable and the amount of GWG during the first trimester is expected to be small, exposure misclassification should be minimal and non-differential with respect to the outcomes. Secondly, since gestational age was estimated based on self-reported LMP, recall errors on the timing of LMP may lead to misclassification on any outcome that was defined based on gestational age. However, since LMP was assessed at the study baseline prior to the exposure assessment, outcome misclassification due to errors on LMP reporting would be non-differential with respect to the exposure, thus diluting the associations. Finally, although our study was relatively large compared to other GWG studies in SSA, our stratified analyses were underpowered. Since the associations between GWG and pregnancy outcomes differ by pre-pregnancy BMI status, and women with lower pre-pregnancy BMI are at particularly higher risk for many pregnancy outcomes [34, 35], future studies are needed to examine the association between GWG and pregnancy-related outcomes among underweight women in SSA. Findings from the present study may be generalized to urban African women with overall good health status. Studies among diverse African populations and by regional status (i.e., urban, rural) are needed to confirm our findings.

## Conclusions

Both inadequate and excessive GWG were associated with higher risks of adverse pregnancy outcomes in African women. Clinical guidelines on GWG should be developed in prevention of both inadequate and excessive GWG, particularly in SSA countries with rising trends of obesity. Intervention trials are warranted to explore effective strategies on GWG management and assess their impacts on preventing adverse pregnancy outcomes among African women.

## Supplementary Information

**Additional file 1: Table S1.** Associations between GWG by percentage adequacy 834 and adverse pregnancy outcomes. **Table S2.** Associations between GWG by z-score and adverse pregnancy outcomes among 854 women with normal BMI at 855 the end of the first trimester.

## Data Availability

The datasets generated and/or analyzed during the current study are not publicly available due to regulatory obligations of the collaborating institutions but may be available from the corresponding author on reasonable request.

## Abbreviations

BMI: Body mass index
CI: Confidence interval
GWG: Gestational weight gain
IOM: Institute of Medicine
LBW: Low birth weight
LGA: Large for gestational age
LMP: Last menstrual period
OR: Odds ratio
SSA: Sub-Saharan Africa
SGA: Small for gestational age
RR: Risk ratio
