# Supplementary material for: Gestational weight gain during the second and third trimesters and adverse pregnancy outcomes, results from a prospective pregnancy cohort in urban Tanzania
Source: Reprod Health. 2022 Jun 16;19:140. doi: 10.1186/s12978-022-01441-7 (PMC9204988; doi:10.1186/s12978-022-01441-7)
Supplement: Supplementary file 1 — Additional file 1: Table S1. Associations between GWG by percentage adequacy 834 and adverse pregnancy outcomes. Table S2. Associations between GWG by z-score and adverse pregnancy outcomes among 854 women with normal BMI at 855 the end of the first trimester. [file 12978_2022_1441_MOESM1_ESM.docx]

**Supplement Table 1.** Associations between GWG by percentage adequacy and adverse pregnancy outcomes

|  |  | **Pregnancy outcomes, risk ratio (95% CI)**^a^ | | | |
| --- | --- | --- | --- | --- | --- |
|  |  | **LBW^b^** | **Preterm birth^c^** | **SGA** | **LGA** |
|  | Case (n, percent) | 92, 7.5% | 195, 15.9% | 199, 16.2% | 134, 10.9% |
| **GWG adequacy^d^** | Inadequate GWG | 1.93 (1.03, 3.63) | 0.86 (0.63, 1.16) | 1.53 (1.14, 2.07) | 0.53 (0.38, 0.77) |
|  | n=553, 45.0% |  |  |  |  |
|  | Adequate GWG | Ref (OR=1.00) | Ref (RR=1.00) | Ref (RR=1.00) | Ref (RR=1.00) |
|  | n=377, 30.7% |  |  |  |  |
|  | Excessive GWG | 1.98 (0.94, 4.16) | 1.25 (0.90, 1.74) | 1.18 (0.80, 1.74) | 1.03 (0.72, 1.47) |
|  | n=300, 24.4% |  |  |  |  |

Abbreviations: gestational weight gain (GWG), low birth weight (LBW), small for gestational age (SGA), large for gestational age (LGA), odds ratio (OR), risk ratio (RR), confidence interval (CI)

^a^ Multivariable model was adjusted for age (years), baseline gestational age (weeks), gestational age at delivery (weeks), BMI at 14 weeks of gestation (underweight, normal, overweight, obese), primigravida status (yes, no), treatment status (iron, placebo), marital status (married, other than married), education (0-4 years, 5-7 years, 8-11 years, ≥12 years), occupation (unemployed, unskilled or informal, skilled), and history of prior complications (yes, no).

^b^ Model for estimating RR did not converge due to small number of LBW events; OR from multivariable logistic regression was reported to approximate RR instead.

^c^ Gestational age at delivery was not adjusted in the model for preterm birth.

^d^ Methods of deriving GWG percentage adequacy and the cutoffs were described in Adu-Afarwuah, Seth, et al. "Maternal supplementation with small-quantity lipid-based nutrient supplements compared with multiple micronutrients, but not with iron and folic acid, reduces the prevalence of low gestational weight gain in semi-urban Ghana: a randomized controlled trial." *The Journal of nutrition* 147.4 (2017): 697-705.

**Supplement Table 2.** Associations between GWG by z-score and adverse pregnancy outcomes among women with normal BMI at the end of the first trimester^a^

|  | **Pregnancy outcomes, risk ratio (95% CI)^b^** | | | |
| --- | --- | --- | --- | --- |
|  | **LBW**^c^ | **Preterm birth**^d^ | **SGA** | **LGA** |
| Case (n, percent) | 66, 8.7% | 124, 16.4% | 140, 17.2% | 82, 10.9% |
| Adequate GWG  (within +/-2 units of GWG z-score) (n=428, 56.7%) | Ref (OR=1.00) | Ref (RR=1.00) | Ref (RR=1.00) | Ref (RR=1.00) |
| Inadequate GWG  (< -2 units of GWG z-score)  (n=327, 43.3%) | 0.77 (0.42, 1.42) | 0.74 (0.53, 1.03) | 1.28 (0.95, 1.72) | 0.78 (0.52, 1.16) |

Abbreviations: body mass index (BMI), gestational weight gain (GWG), low birth weight (LBW), small for gestational age (SGA), large for gestational age (LGA), odds ratio (OR), risk ratio (RR), confidence interval (CI)

^a^ One participant with normal BMI had GWG z-score above 2 units and therefore was excluded from analysis (total n=755).

^b^ Multivariable model was adjusted for age (years), baseline gestational age (weeks), gestational age at delivery (weeks), BMI at 14 weeks of gestation (underweight, normal, overweight, obese), primigravida status (yes, no), treatment status (iron, placebo), marital status (married, other than married), education (0-4 years, 5-7 years, 8-11 years, ≥12 years), occupation (unemployed, unskilled or informal, skilled), history of prior complications (yes, no).

^c^ Model for estimating RR did not converge due to small number of LBW events; OR from multivariable logistic regression was reported to approximate RR instead.

^d^ Gestational age at delivery was not adjusted in the model for preterm birth.
